# Cefepime-induced neurotoxicity in critically ill patients undergoing continuous renal replacement therapy: beware of dose reduction!

**DOI:** 10.1186/s13054-015-1179-z

**Published:** 2015-12-30

**Authors:** Patrick M. Honore, Herbert D. Spapen

**Affiliations:** ICU Department, Universitair Ziekenhuis Brussel, Vrije Universiteit Brussel, Brussels, Belgium

The recently published article by Fugate et al. [1] reminds us of the frequently underappreciated yet deleterious neurotoxic potential of cefepime (CEF) in critically ill patients with pre-existent or acute kidney dysfunction [2]. We do agree with the authors that dose adjustments for renal function are critical to minimize the risk of CEF neurotoxicity. However, we would like to warn of daredevil CEF dose reduction in patients undergoing continuous renal replacement therapy (CRRT). Indeed, approximately one-fifth of the patients studied by Fugate et al. were treated with CRRT and most of them received less than 1 g CEF every 12 h [1]. CEF has a very low protein binding and distribution volume and thus will easily be removed by CRRT [3]. Seyler et al. previously studied whether recommended CEF doses (2 g, twice daily) enabled an appropriate pharmacokinetic/pharmacodynamic (PK/PD) target concentration to be reached for treatment of *Pseudomonas aeruginosa* (32 mg/L, corresponding to four times the EUCAST minimal inhibitory concentration (MIC)) in patients undergoing CRRT. They observed that none of the patients attained the suggested PK/PD target within the first 48 h of treatment, even when a 2 g CEF bolus dose was given [4]. According to Seyler et al.’s results, the applied CEF regimen would allow 90 % coverage of micro-organisms with a MIC ≤2, implicating that less susceptible bacteria escape treatment. Thus, applying a CEF dose of 2 g or less during CRRT, as suggested by Fugate et al., may decrease the risk of CEF-induced neurotoxicity at the cost of inducing treatment failure or resistance! We strongly argue against dose reduction of CEF during CRRT and even prescribe higher doses (2 g, three times daily) in CRRT patients. Close monitoring of neurotoxicity, preferably using 24-h recording of electrical brain activity, remains imperative. Therapeutic drug monitoring has been advocated but its validity to support dose adaptation of β-lactam antibiotics is questionable and any dose relationship between CEF levels and occurrence of neurotoxicity remains to be proven. If neurotoxicity is detected, other antibiotics (e.g., colistin) must be administered.

## Abbreviations

CEF: Cefepime
CRRT: Continuous renal replacement therapy
MIC: Minimal inhibitory concentration
PK/PD: Pharmacokinetic/pharmacodynamics
